# Exploring registers of valuing flat glass recycling in the construction sector: A case study in Sweden

**DOI:** 10.1177/0734242X251415075

**Published:** 2026-02-12

**Authors:** Malin Nilsson, Wiktoria Glad, Maria Eidenskog

**Affiliations:** 1 Department of Thematic Studies, Linköping University, Linköping, Sweden

**Keywords:** Flat glass recycling, construction sector, qualitative research, case study, workshop, interviews, registers of valuing, Sweden

## Abstract

Rapid urbanisation and population growth have increased the demand for glass as a building material, but its environmental friendliness is questioned partly due to the lack of recycling. The production of flat glass is energy-intensive and contributes to significant greenhouse gas emissions, while the raw materials used are finite resources. This research article explores the challenges and opportunities for recycling flat glass in the construction sector in Sweden. Despite its ability to be endlessly recycled without degradation, flat glass is rarely recycled and often ends up in landfills. Barriers to circular handling of flat glass include economic imbalances, high transportation costs and lack of collection and sorting facilities. The research was conducted using a qualitative case study approach, including a workshop and semi-structured interviews, to identify registers of valuing flat glass recycling. The results highlight the different roles of glass, its economic and ecological values and the tensions between different registers of valuing glass. Understanding these values and tensions can contribute to more precise measures for integrating flat glass into a circular economy and valorisation of post-consumer flat glass.

## Introduction

Rapid urbanisation and population growth are fuelling an eruption in demand for glass as a building material (Butler and Hooper, 2019; Delbari and Hof, 2024). Flat glass comes with many attributes that make it attractive in the construction sector. It is described as a sustainable, environmentally friendly, resilient, readily available and comparatively cheap material (Achintha, 2016). The transparent quality of glass makes it appealing to work with since it brings value to the indoor environment by blurring the line between nature and the anthropogenic (Achintha, 2016; Arbab and Finley, 2010; Souviron et al., 2019; Westbroek et al., 2021). Flat glass is also a commodity that theoretically could be endlessly recycled without being downgraded; however, in practice, contamination, complex material composition and a lack of infrastructure and quality guarantees are some of the factors that limit glass recycling (Butler and Hooper, 2019; Delbari and Hof, 2024; Popa et al., 2018). The description of flat glass as environmentally friendly is thus ambiguous at best, seeing that the material is not being recycled in the contemporary construction sector (Achintha, 2016; Billore, 2021; Geboes et al., 2022). More often, post-consumer flat glass obtained from renovating or demolishing buildings is crushed together with other building materials and sent to landfills (Hestin et al., 2016). The process of proper handling of glass during renovation and demolition is complex and small mistakes can lead to contamination, which renders the flat glass unrecyclable. Typically, different kinds of flat glass are present at a renovation/demolition site; these all need to be sorted, contained and transported separately (Billore, 2021; Larsen et al., 2012).

Furthermore, flat glass production is an energy-intensive industry and contributes to significant global greenhouse gas emissions (Westbroek et al., 2021). This is due to the high temperature of 1500°C needed to melt the raw materials in the furnace to produce molten glass (Del Rio et al., 2022; Hartwell et al., 2023; Westbroek et al., 2021). The continued extraction of raw materials, silica sand, soda ash, limestone, soda feldspar and dolomite, also contributes to environmental impacts from the float-glass industry since they are finite resources that will run out (Del Rio et al., 2022; Hartwell et al., 2023). The environmental impacts of glass as a building material expand further since a sheet of flat glass is rarely used as it is (Souviron et al., 2019). Instead, complex material composition often makes glass difficult to recycle and reuse (Delbari and Hof, 2024). Reuse of flat glass is preferred to recycling for environmental reasons, but comes with significant challenges, such as technical requirements for modern glass (safety, energy, noise, sunshades), and the lack of certification systems to guarantee quality and safety (Billore, 2021). There are also different levels of recycling where ‘open-loop recycling’ reuses waste glass in new, often lower-grade applications, while the preferred ‘closed-loop recycling’ turns waste glass back into the same type of glass, preserving its value and closing the material loop (Yuan et al., 2024).

Glass from renovations and demolitions of buildings can be used to reduce the climate impact from both the flat glass manufacturing industry and the building sector (Achintha, 2016; Del Rio et al., 2022; Hartwell et al., 2023; Tihomirovs, et al., 2023; Westbroek et al., 2021). However, an accompanying challenge lies in determining where these climate benefits should be attributed, as assigning them to one sector or sharing them across both has implications for policy, incentives and sustainability reporting. The construction industry benefits from reduced landfill and resource depletion, while the glass manufacturing industry gains large reductions in energy use and emissions when recycled cults replace virgin raw materials (Yuan et al., 2024).

Moreover, it seems that the construction sector has entered a path-dependence where flat glass is handled as waste, creating a trajectory for glass ending up in landfills (Hestin et al., 2016; Souviron and Khan, 2020). Post-consumer flat glass could enter a circular economy, but it is a nebulose process surrounded by complex circumstances (Achintha, 2016; Billore, 2021; Delbari and Hof, 2024; Forslund and Björklund, 2022). In addition, the multiple understandings of *circular economy* add to the complexity (Brandão et al., 2020; Geissdoerfer et al., 2017; Ghisellini et al., 2016).

From this background, there is a need to further understand different actors’ valuation of glass, the practices of handling glass and how these aspects relate to a potential circular flow of flat glass. To understand the multiple ways glass is used and discussed in different settings and by different actors, this article will explore glass with registers of valuing (Abrahamsson, 2019, 2022; Beltrame, 2019; Gallisti, 2020; Hammarfelt et al., 2020; Heinich, 2021; Heuts and Mol, 2013; Lamberg et al., 2023; Lehtonen and Pyyhtinen, 2020; Schrøder et al., 2022) aiming to ‘capture the enactment of different practices of valuation in concrete situations in order to assess value’ (Beltrame, 2019: 176). Valuing is something our informants ‘do’ as activities part of their professional practices (Heuts and Mol, 2013). When researchers analyse these practices, different registers of valuing surface; some are separate, some overlap and some create tensions. Registers vary from case to case but could, for example, be aesthetics, handling, historical references, monetary, non-renewable resource use, etc. This article aims to understand the practices and the registers of valuing related to flat glass recycling. Making the different ways flat glass is valued and handled in practice will enable a deeper understanding of the challenges involved in implementing a circular flow of flat glass.

Previous research highlights several barriers that hinder circular handling of flat glass in the construction sector. Some that have already been addressed include the hardship of breaking the path-dependency and ‘business-as-usual’, viewing end-of-life glass as waste or deeming glass that still has efficient properties as end-of-life waste (Hestin et al., 2016; Souviron and Khan, 2020). However, there are multiple roadblocks on the way to entering flat glass in a circular loop, ‘[. . .] there is a serious lack of sustainable consumption and management systems that can support the recycling and reusing of flat glass’ (Billore, 2021: 117). The low support and the lack of a system for recycling and reusing flat glass are aspects of the difficulty to come unstuck from this path-dependency of treating post-consumer flat glass as waste (Zarrinpoor, 2021).

One barrier widely acknowledged in previous research is the economic circumstances surrounding post-consumer flat glass recycling (Billore, 2021; Souviron et al., 2019; Yuan et al., 2024). This is due to the economical imbalance between post-consumer flat glass recycling and ‘business-as-usual’, that is, the material ending up in landfills. Hestin et al. (2016) highlight four main areas that contribute to this imbalance: (1) the low use of post-consumer flat glass in flat glass manufacturing, (2) high transportation costs, (3) low landfilling prices and (4) difficult-to-access collection and sorting facilities.

According to Hestin et al. (2016), it is impossible to analyse the opportunities of recycling flat glass on an international scale without first scrutinising the capacity for implementation on a local and regional scale. This is due to differences in ‘business-as-usual’ between countries and national and regional legislations regarding waste management. Firstly, the system needs to be in place regionally and nationally to expand said system (Hestin et al., 2016).

Following this argument, this article is delimited to Sweden with extensions to neighbouring countries through international organisations. In 2021, only 1% of post-consumer flat glass was recycled in Sweden, but recently, initiatives for organising recycling processes are growing, making it an interesting site of study.

## Method

This research was conducted with a qualitative case study approach (Stake, 2010) including data collection through a workshop (Eidenskog et al., 2024; Ørngreen and Levinsen, 2017; Roos and Nilsson, 2020) and semi-structured interviews (Brinkmann and Kvale, 2018; Galletta, 2013). Qualitative research is constrained in replicability but provide in-depth understanding of problems. Workshops have recently gained traction in academic research and promise a focused but still creative way to collect data (Eidenskog et al., 2024). Our workshop included 18 participants of interest for the circular economy of flat glass (see Table 1), interesting topics were identified, which further guided the methodological approach. The research gaps were strengthened by reviewing previous research to invigorate the research design (Galletta, 2013). This resulted in 11 semi-structured interviews with 12 commercial manufacturers and consumers of flat glass. These were, that is, architects, flat glass manufacturers, construction companies, property managers, architecture consultants and flat glass wholesalers (see Table 1).

**Table 1.** Participants in empirical data collection.

Type of organisation	Participant/job title	Workshop participation	Semi-structured interview
FGM Poland	Flat glass innovation team	✔	✔
FGM Poland	Technical specification manager	✔	✔
The PA	Sustainable specialist	✔	✔
FGIA	Project manager sustainability	✔	✔
FGW	Sustainable specialist	✔	✔
PM	Sustainable specialist	✘	✔
ATC	Sustainable consult	✘	✔
AC	Architect/Sustainability specialist	✔	✔
FGRF	CEO	✔	✔
CC	Environmental coordinator	✘	✔
CC	Sustainability specialist	✘	✔
MHC	Energy and environmental strategist	✔	✘
WGRC	CEO	✔	✘
RI	Senior project manager	✔	✘
FGM France	R&D manager for the recycling of flat glass	✔	✔
FGRF	Adviser	✔	✘
RC	Project manager	✔	✘
RC	Business project manager	✔	✘
ORC	President	✔	✘
FGM Nordic and Baltic	Purchasing category manager	✔	✘
RI	Senior associate professor	✔	✘

FGM: flat glass manufacturer; PA: procurement authority; FGIA: flat glass industry association; FGW: flat glass wholesaler; PM: property manager; ATC: architecture and technology consultant; AC: architectural company; FGRF: flat glass recycling firm; CC: construction company; MHC: municipal housing company; WGRC: window glass recycling company; RI: research institute; RC: recycling company; ORC: organisation for recycling companies.

### Workshop design

The workshop focused on how the implementation of a circular flow of flat glass can provide value for the actors involved in the project. The aim of the workshop was to gain insight into the actors’ practices on the issues and roadblocks hindering the recycling of flat glass within the construction sector. The workshop was designed to both bring new ideas to the participants as well as provide space for discussions and creative activities. The first part of the workshop included a 15-minute presentation of current research on the implementation of new technologies and how to create engagement throughout organisations. We advised the participants to think about how to create broad engagement in their organisations, what possible actors they could enrol or who in their organisations could be a good spokesperson for the change in their work practices.

We then divided the participants into three groups with four to six participants and gave everyone a large paper sheet, colour pencils and crayons. Working with unfamiliar tasks in workshops, such as drawing, has the advantage of stimulating creativity and spurring new conversations (Cornwall and Jewkes, 1995; Stadil and Tanggard, 2014). We gave the instructions to the participants to draw a ‘road map’ for their organisation when it came to a circular flow of flat glass. The only rule in this exercise was to avoid using words and instead try to visualise the path forward with pictures. Each group was observed by a researcher who, in a few instances, commented on the discussions or asked follow-up questions. After silently drawing by themselves, the participants were asked to describe their maps to each other.

The discussions required very little moderation and the participants freely discussed each other’s drawings. After these discussions, we had a large group discussion where each group raised some points they found particularly interesting. When we introduced the task of the roadmap, we asked the participants who wanted to contribute to our research to leave their drawings behind. Most participants happily left us their drawings to be used in the research project. The drawings were analysed using a visual brainstorming method where the researchers searched for common themes and gaps that could be addressed in the interviews.

### Semi-structured interviews

The workshop sparked an interest in the topic of values surrounding flat glass. Rooted in socio-material relations and our theoretical approach questions regarding different values of glass as a material arose. To explore this domain further, a semi-structured interview approach was applied to the study. The first step towards conducting the interview was constructing an interview guide adapted to the respondents.

The questions involved the search for a deeper understanding of the participants’ perception of, that is, values within circular handing of post-consumer flat glass. These questions, along with a review of previous research concerning circular handling of post-consumer flat glass, formed a preliminary interview guide. Questions were formed to be flexible, open-ended and participant-oriented, seeing as the participants came from a variety of background in the construction sector. The interview guide was then tested through a follow-up interview with one of the participants of the workshop to confirm the relevance of the questions. After the pilot-interview small changes were made by re-formulating questions, refining follow-up questions and rearranging order.

All interviews were conducted digitally using Teams video call, the duration varied between 45 and 90 minutes; however most interviews were carried out in approximately 60 minutes. Interviews were carried out in Swedish, English or Norwegian depending on the informants’ preferred language. The interviews were recorded, and transcriptions were conducted continuously during the interviewing phase.

### Qualitative content analysis

Following the transcription of the interviews, the analysis of the data began. To understand the registers of valuing glass within the interviews, a qualitative content analysis was applied. Further, to enhance the understanding of the data, the interviews were coded into categories with the aim of creating a condensed inference of the interviews into the context of our study (Neuendorf, 2017). Nvivo14 was used to conduct the coding.

An initial open coding was conducted during the transcription phase where notes were taken, and potential categories were formed. This resulted in three head categories generated before the main coding (see Table 2). The three categories were generated both directly from the empirical data and the theoretical approach of this study. During the second reading of the interviews the main coding began, and subcategories were formed freely during the coding process (Neuendorf, 2017). This phase consisted of interpreting the data from the interviews into the categories. This continued until the categories were saturated, meaning that no more data was interpreted from the interviews.

**Table 2.** Categories and participants’ answers (no.) in qualitative content analysis.

Circular value network		The value of glass		What is glass	
Sub-categories	Participants (no.)	Sub-categories	Participants (no.)	Sub-categories	Participants (no.)
Responsibility	FGM (3), CC (2), FGIA (1), FGW (1), AC (1), ATC (1), PM (1), FGRF (1)	Ecological	FGM (2), FGW (1), FGIA (1), AC (1), ATC (1), FGRF (1)	What is enacted	FGM (3), CC (2), FGW (1), FGIA (1), PA (1), AC (1), PM (1), FGRF (1)
Demand	FGM (3), CC (2), FGIA (1), FGW (1), PA (1), AC (1), ATC (1)	Economic	FGW (1), FGIA (1), PA (1), AC (1), ATC (1)	Other versions of reality	FGM (3), CC (2), FGW (1), FGIA (1), PA (1), PM (1), FGRF (1)
Knowledge	FGM (3), CC (2), FGIA (1), FGW (1), PM (1), AC (1), ATC (1), PM (1), FGRF (1)	Functional	FGM (2), FGIA (1), PA (1), AC (1)		
Legislations	FGM (3), FGIA (1), FGW (1), ATC (1), WGRC (1)	Other values	FGM (1), FGW (1), FGIA (1), PA (1)		
Logistics	FGM (3), FGW (1), FGIA (1), PA (1), FGRF (1)				
Cooperation	FGM (3), CC (2), FGIA (1), PA (1), AC (1), ATC (1), FGRF (1)				
Difficulties	FGM (3), CC (2), FGW (1), FGIA (1), PA (1), AC (1), ATC (1), PM (1), FGRF (1)				
Reuse	FGM (1), FGW (1), FGIA (1), AC (1), ATC (1), PM (1), FGRF (1)				

FGM: flat glass manufacturer; PA: procurement authority; FGIA: flat glass industry association; FGW: flat glass wholesaler; PM: property manager; ATC: architecture and technology consultant; AC: architectural company; FGRF: flat glass recycling firm; CC: construction company; WGRC: window glass recycling company.

## Results

### Enactments of glass

Glass takes on multiple forms in our empirical data, from descriptions rooted in its attributes as a building material to deeper, more abstract meanings behind the entity that is glass. What glass is, is a broad question, and so are the enactments of the material by the participants in this study. The respondents describe glass as a mundane material, transparent, invisible, there but never to be noticed, taken for granted and appointed as waste after consumption. However, some descriptions are more colourful, describing glass as important, enriching, comforting and remarkable in bringing many positive properties into everyday life.

The view of glass as a mundane material is depicted by respondents as the general understanding of what glass is. The transparent quality of the material respecifies it as invisible in everyday life, and as a result, the values it provides become invisible. One respondent expressed that:

‘[. . .] most of the people don’t know that glass is so important because, can you imagine our buildings without windows [. . .]. It’s so obvious that glass is everywhere, but nobody thinks about it’ (Greta, FGM).

Here, glass is taken for granted, not thought of as a contributor in everyday life. However, respondents express the importance of windows and that ‘you think of windows as frames with glass, you don’t think of glass as a separate material’ (Gustav, PA). Once more, glass is made invisible through the conversion into windows. When glass is put into a frame and becomes a window, it loses its properties as a material and is no longer viewed as glass. It is instead understood, valued and seen as a window. One respondent stated that ‘glass means nothing’ (Göran, AC) when asked about the value of glass as a material in their profession as an architect, but highlighted the importance of windows for, that is, making boundaries between the outside and the inside.

Respondents also enacted glass as dangerous. One respondent working as a flat glass wholesaler stated that ‘I think every person and everyone, I hire in this company starts with an inherent fear of glass’ (Gösta, FGW). This inherent fear of glass and injuries related to the material can, according to multiple respondents, create a hesitance in working with glass. Here, glass is enacted through its dangerous attributes.

Further, glass is enacted through its functional attributes. Here, glass is viewed as important, valuable and one of a kind. One respondent, working within flat glass manufacturing, described glass as:

[. . .] pretty remarkable because as we speak it is still the only material that is able to provide daylight and comfort while being able to sustain a building for 40-50 years, which makes it quite remarkable because for all other materials in a building there are other alternatives that exist for glass, there are none for the moment (Gunnar, FGM).

This view contradicts the conversion of glass into windows and acknowledges that it is the material itself that brings the attributes of functional values. The description of glass as remarkable is strengthened by a project manager for a flat glass association expressing that:

[. . .] there is so much you can build into glass; it can be sound protection, it can be light protection, it can be sun protection, solar cells, you name it, and you can do it with glass. There are few materials that are so multifunctional, so I would say that the material gives so many more attributes than just being glass. (Gudrun, FGIA)

Through this enactment of the material glass becomes uplifted as something more than just the material; it is irreplaceable in relation to the functional values it brings into the construction sector.

Then, what is glass? We can see that the material is enacted differently depending on the actor’s relation to the material. According to respondents, the overarching enactment of glass in everyday life is as an invisible and mundane material. Registers of valuing what glass is entail the register of functional values, its dangerous attributes and the conversion of the material into windows in the construction sector.

### Post-consumer flat glass enacted in a circular economy

The topic of valuing glass was added in the interviews because of questions that arose during the workshop, where the economic value was at the central register of valuing. The main challenge highlighted by participants in the workshop was the fact that glass is cheap to dispose of and most often ends up in landfills. Besides the economic aspect, actors also expressed a concern regarding other registers of valuing flat glass. These values were, for example, connected to the view of flat glass as ‘trash’ hence the attributes of the post-consumer material are not highly valued. Further, it was expressed that there is low economic value in post-consumer flat glass, contributing to the low overall value of the material. Through the analysis of the interviews, multiple registers of valuing were enacted.

The main registers of valuing enacted in the interviews were ecological and economic aspects regarding circular handling of post-consumer flat glass. Different registers of valuing were raised concerning the point at which flat glass should be valued as end-of-life. Respondents in the network positioned further away from the construction sector, that is, flat glass manufacturers and wholesalers, were less prone to the reuse of flat glass in new buildings and primarily advocated for recycling. Respondents situated within the construction sector pressed that windows need to be reused until end-of-life to reduce the production of flat glass, which was described as an energy and emission-intensive business. The register of valuing is divided in the sense that stakeholders working with flat glass manufacturing value the post-consumer material to use in their production. Stakeholders in the construction business, on the other hand are open for the register of valuing the material until it is deemed ‘unusable’ and thus at the end-of-life.

Respondents also touched on the register of valuing flat glass manufactured with recycled glass. One respondent, working as a research and development manager for recycling of flat glass, asked himself: ‘What are the benefits as a consumer of buying a window that has 90 percent recycled content?’ (Gunnar, FGM) As an answer to this self-exploratory question the respondent expressed that:

Honestly, if you only look at the performance there are no benefits, it is only our own belief that it is the right thing to do, but it costs 20 percent more [. . .]. It is about us thinking that it is a good thing to do, and we are okay with paying more because we know it is the only way to sustain the world in the long run [. . .]. (Gunnar, FGM)

In this view, the economic and ecological registers of valuing are being pitted against each other, which is a recurring approach among the respondents. In this specific example, the economic burden of investing in flat glass manufactured with recycled materials can only be achieved through a switch in the mindset of the consumer.

Using flat glass manufactured from recycled glass in construction is more expensive and the respondent voices a concern for a price-based mindset because of the lack of added value. This is further strengthened by the statement that:

The main barrier is, as usual cost, [. . .] it is the real difficulty because it is an extra cost. So, in a way, if there is an extra cost, there must be an extra value. To me, cost and value are similar, if there is added value, the cost is not so much of an issue in the end, it is a matter of extra value and there’s the bigger mindset. For me, if the mindset changes the rest will follow. (Gunnar, FGM).

The lack of added value was also portrayed by an architect, stating that:

If you’re going to make a window, it’s glass, there are no options, glass made of what? It’s glass. [. . .] Of course, you think a lot about the properties of the glass, but we do not think about if it is recycled glass or not. It’s more about what kind of floor we should have, we want natural stone, it’s too expensive, we use terrace flooring instead, but no, it has concrete in it, so it will have a high climate impact. It speaks volumes because with other materials, you have several options to consider, but a window is a window. (Göran, AC)

This respondent acknowledges the lack of value in using windows manufactured with recycled glass while still pointing out that other materials have options with lower climate impact. Hence, there is a lack of registers of valuing both the post-consumer flat glass and the product if used to manufacture.

The ecological register of valuing post-consumer flat glass enacted by the respondent’s is mostly tied to emission mitigation in connection to the manufacturing of flat glass. A respondent working with flat glass manufacturing expressed that using post-consumer glass in the manufacturing process ‘is the only way to a carbon dioxide neutral glass production [. . .]’ (Greta, FGM) All respondents working with flat glass manufacturing agreed that the emission mitigation achieved by returning post-consumer flat glass into the manufacturing process is valuable in the circular flow of the glass. Though ecological registers of valuing post-consumer flat glass are not exclusively tied to emission mitigation. It was also expressed that ‘[. . .] there is a value in circular handling of flat glass considering that we are saving nature and reducing the environmental burden [. . .].’ (Gottfrid, FGRF) This adds another dimension to the register of valuing glass through ecological values, where respondents highlight the environmental benefits of reducing the use of raw materials in flat glass manufacturing.

Respondents agree that ecological aspects in the circular flow of flat glass could be a driver for change if there were a.) more knowledge about the environmental impact of glass production among stakeholders in the construction sector and b.) a general understanding of the ecological benefits of recycling flat glass. Awareness regarding the climate impact of glass ending up in landfill is low among decision makers in the construction sector. Ultimately, respondents raised concern for the clash between economic register of valuing versus ecological register of valuing in the circular flow of post-consumer flat glass and the need for equality between the two. When talking about the economic circumstances surrounding post-consumer flat glass recycling, a respondent expressed that ‘[w]e would need to have an economic value in glass, at the moment it is just a cost [. . .] the economic sustainability does not exist to be ecologically sustainable’. (Gösta, FGW) The impact the economy has on the circular flow of post-consumer flat glass is depicted as problematic and there is a worry among respondents that the inherent lack of economic value of the material will continue to create difficulties on the road towards circularity. Overall, there are several embedded issues connected to the economic register of valuing within post-consumer flat glass recycling. There was a consensus amongst respondents that the economic situation creates a locked state for post-consumer flat glass, where alternative processes are hindered by a mindset fixated on cost.

### Tensions in the circular flow of post-consumer flat glass

Similar to Lamberg et al. (2023), we found tensions between different registers of valuing, in our case between economics, ecological, space-timing and knowledge. The most prominent tension enacted was between the registers of economics and ecological valuing. As mentioned in the previous section, the register of ecological valuing is pitted against the economic register of valuing. This created a clash within the circular economy of post-consumer flat glass seeing as the economic register is prioritised over the register of ecological valuing. It also creates complexity as there are multiple layers enacted within the register of valuing flat glass through cost and economics, thus creating internal tension within the register itself. There are two prominent ways in which the economic register of valuing is enacted in the interviews (a) the lack of economic value of flat glass and (b) the high cost of recycling post-consumer flat glass. Both enactments contribute to tension within the circular economy of the post-consumer material.

Differences in registers of valuing glass as end-of-life create tension within the circular value network. The actors’ position within the circular value network mattered and what action would be most valuable for them. Respondents working with flat glass manufacturing express a need for post-consumer flat glass in their manufacturing to produce flat glass with a high content of recycled material. Hence, recycling post-consumer flat glass is enacted as more valuable than reuse by these actors. The Swedish construction sector works through the EU directive of waste hierarchy, where the reuse of materials is classified as a better option for the environment than recycling. Hence, the first alternative will always be reusing post-consumer flat glass before recycling it, if it is not sent to landfill (cf. Delbari and Hof, 2024). These different registers of valuing glass could work against the implementation of a circular flow of flat glass.

Further, there is tension between actors concerning the ecological register of valuing. Respondents express that actors within flat glass manufacturing see the importance of recycling glass while it is hard to engage stakeholders in the construction sector, that is, the decision-makers in the handling of post-consumer flat glass. This is partly due to the economic register of valuing, as presented earlier, but also because actors in the construction sector cannot account for the environmental benefits. The environmental values are more visible to actors within flat glass manufacturing because it helps their work towards climate neutrality while actors in the construction sector cannot include the carbon reductions of recycling post-consumer flat glass in their sustainability reporting. One respondent, working as a sustainability specialist for a construction company expressed that:

[. . .] what I find challenging is that there is a lot of focus on new construction, that you have to build something, and use recycled or reused materials and then you get to credit it to your new building, but there are very few incentives for the building that is to be demolished or dismantled. (Gertud, CC)

Hence, there is a need for more incentives that give credit to actors within the construction sector for recycling post-consumer flat glass. This would reduce the tension within the ecological register of valuing.

Time-spacing of the recycling process of post-consumer flat glass to new flat glass and not degrade the glass to containers or mineral wool insulation is a key concern for the recycling industry (Stålhandske et al., 2024). Handling the flat glass with care from the demolition site to the only recycling station in Sweden forms part of the economic register of valuing: ‘[. . .] the process longer, a little bit more difficult, a little bit more expensive than the regular way’ (Gunnar, FGM). Several interviewees and workshop participants mentioned the logistical difficulties and the balancing of the landfill costs and costs for recycling: ‘I think that it is not the flat glass recycling process itself that is too expensive but rather landfill that is too cheap’ (Gunborg, Construction company)

The register of valuing, including the recycling alternative as more costly, seems to be more prominent for actors with more physical distance from the construction industry. The distance could give a better overview of how recycling of flat glass is valued more generally, or the perception could be based on older information and the actors in the construction industry are more up to date: ‘[. . .] there are a lot of preconceived notions and myths such as that it takes much longer and it will be more difficult and expensive [. . .]’ (Gert, PM). The knowledge register of valuing includes the communication of recycling of flat glass, how it is presented, in what words and by whom. Architects, builders, property owners and recycling management professional staff are all part of a chain where communication could be improved to bridge knowledge gaps about recycling of flat glass.

## Conclusions

This paper set out to understand different registers of valuing tied to post-consumer flat glass recycling and professional actors. We aimed to provide insights into challenges in creating a closed circular flows of flat glass to avoid degradation to material for food containers, mineral wool for insulation or sending the glass to landfill. Our analyses showed how the valuing of post-consumer flat glass included ecological, economic, time-space and knowledge registers and how tensions were present between registers of valuing.

Flat glass is understood as both mundane and extraordinary, being the most long-lasting construction material in buildings. The multi-functionality of glass provides basic needs for sheltering, aesthetic qualities to enhance building designs and versatility in size, shape, and colour. Like other market products, glass has also been diversified as a response to construction sector demands and innovations in glass manufacturing methods, making recycling more difficult as the complex material composition risk contaminating the cullet. Glass was also enacted as dangerous during demolition of buildings and must be handled with care for humans and materials in the recycling process (cf. Lehtonen and Pyyhtinen, 2020).

The registers of valuing flat glass recycling came with tensions between ecology and economy, time-space constraints and perceived knowledge gaps. The registers were enacted in practices covering different parts of the value chain of flat glass: from the planning and design by architects, developers and construction companies, builders and demolition businesses, property owners, flat glass manufacturers, interest organisations and recycling companies. Tensions between different practices in the value chain were also present. Practices by architects in the design phase will have consequences years later for the companies involved in demolition and recycling of flat glass. The decisions made by architects on sizes, shapes and complex material composition of flat glass will influence the recyclability several decades later. The many different types of glass will make recycling more difficult but might have aesthetic values for users of the buildings. Reuse is preferred before recycling, if we follow the European Union waste hierarchy, but the glass manufacturers are naturally inclined to recycling, which is yet another tension in the circular economy of flat glass.

Through our theoretical approach based in registers of valuing (Heuts and Mol, 2013) different enactments of flat glass and recycling of the material became visible. The enactments were associated with the different roles and organisations of the participants, making flat glass and recycling socio-material matters that could not be separated but must be studied together and in practices. Understanding of practices could provide opportunities for more accuracy in target measures when flat glass becomes part of the circular economy. Acknowledging tensions in processes for bringing post-consumer flat glass into circular flows will also contribute to a better understanding of how waste materials could be valorised and part of the circular economy.
